# Missense variant in *TREML2* protects against Alzheimer's disease

**DOI:** 10.1016/j.neurobiolaging.2013.12.010

**Published:** 2014-06

**Authors:** Bruno A. Benitez, Sheng Chih Jin, Rita Guerreiro, Rob Graham, Jenny Lord, Denise Harold, Rebecca Sims, Jean-Charles Lambert, J. Raphael Gibbs, Jose Bras, Celeste Sassi, Oscar Harari, Sarah Bertelsen, Michelle K. Lupton, John Powell, Celine Bellenguez, Kristelle Brown, Christopher Medway, Patrick CG. Haddick, Marcel P. van der Brug, Tushar Bhangale, Ward Ortmann, Tim Behrens, Richard Mayeux, Margaret A. Pericak-Vance, Lindsay A. Farrer, Gerard D. Schellenberg, Jonathan L. Haines, Jim Turton, Anne Braae, Imelda Barber, Anne M. Fagan, David M. Holtzman, John C. Morris, The 3C Study Group, the EADI consortium, the Alzheimer's Disease Genetic Consortium (ADGC), Alzheimer's Disease Neuroimaging Initiative (ADNI), the GERAD Consortium, Julie Williams, John S.K. Kauwe, Philippe Amouyel, Kevin Morgan, Andy Singleton, John Hardy, Alison M. Goate, Carlos Cruchaga

**Affiliations:** a Department of Psychiatry, Washington University School of Medicine, St. Louis, MO, USA; b Department of Neurology, Washington University School of Medicine, St. Louis, MO, USA; c Department of Pathology and Immunology, Washington University School of Medicine, St. Louis, MO, USA; d Department of Genetics, Washington University School of Medicine, St. Louis, MO, USA; e Department of Developmental Biology, Washington University School of Medicine, St. Louis, MO, USA; f Knight Alzheimer's Disease Research Center, Washington University School of Medicine, St. Louis, MO, USA; g Hope Center for Neurological Disorders, Washington University School of Medicine, St. Louis, MO, USA; h Institute of Psychological Medicine and Clinical Neurosciences, Cardiff University, Cardiff, UK; i Diagnostic Discovery Department, Genentech Inc, South San Francisco, CA, USA; j Department of Bioinformatics and Computational Biology, Genentech Inc, South San Francisco, CA, USA; k Human Genetics Department, Genentech Inc, South San Francisco, CA, USA; l Department of Molecular Neuroscience, UCL Institute of Neurology, London, UK; m Laboratory of Neurogenetics, National Institute on Aging, National Institutes of Health, Bethesda, MD, USA; n Department of Biology, Brigham Young University, Provo, UT, USA; o Department of Neurology, Taub Institute on Alzheimer's Disease and the Aging Brain, Columbia University, New York, NY, USA; p Gertrude H. Sergievsky Center, Columbia University, New York, NY, USA; q The John P. Hussman Institute for Human Genomics, University of Miami, Miami, FL, USA; r Dr John T. Macdonald Foundation Department of Human Genetics, University of Miami, Miami, FL, USA; s Department of Medicine, Boston University Schools of Medicine and Public Health, Boston, MA, USA; t Department of Biomedical Genetics, Boston University Schools of Medicine and Public Health, Boston, MA, USA; u Department of Neurology, Boston University Schools of Medicine and Public Health, Boston, MA, USA; v Department of Ophthalmology, Boston University Schools of Medicine and Public Health, Boston, MA, USA; w Department of Epidemiology, Boston University Schools of Medicine and Public Health, Boston, MA, USA; x Department of Biostatistics, Boston University Schools of Medicine and Public Health, Boston, MA, USA; y Department of Pathology and Laboratory Medicine, Perelman School of Medicine at the University of Pennsylvania, Philadelphia, PA, USA; z Department of Molecular Physiology and Biophysics, Vanderbilt Center for Human Genetics Research, Vanderbilt University, Nashville, TN, USA; aa Human Genetics, School of Molecular Medical Sciences, University of Nottingham, Nottingham, UK; bb Institute of Psychiatry, King's College London, London, UK; cc Inserm, Lille, France; dd Universite Lille 2, Lille, France; ee Institut Pasteur de Lille, Lille, France

**Keywords:** TREM2, Genome-wide association studies, Conditional analysis, Endophenotype, Gene, Alzheimer's disease, Association

## Abstract

TREM and TREM-like receptors are a structurally similar protein family encoded by genes clustered on chromosome 6p21.11. Recent studies have identified a rare coding variant (p.R47H) in *TREM2* that confers a high risk for Alzheimer's disease (AD). In addition, common single nucleotide polymorphisms in this genomic region are associated with cerebrospinal fluid biomarkers for AD and a common intergenic variant found near the *TREML2* gene has been identified to be protective for AD. However, little is known about the functional variant underlying the latter association or its relationship with the p.R47H. Here, we report comprehensive analyses using whole-exome sequencing data, cerebrospinal fluid biomarker analyses, meta-analyses (16,254 cases and 20,052 controls) and cell-based functional studies to support the role of the *TREML2* coding missense variant p.S144G (rs3747742) as a potential driver of the meta-analysis AD-associated genome-wide association studies signal. Additionally, we demonstrate that the protective role of *TREML2* in AD is independent of the role of *TREM2* gene as a risk factor for AD.

## 1 Introduction

Genome-wide association studies (GWAS) are a very powerful approach for identification of novel loci associated with disease status or other complex traits. However, these single nucleotide polymorphisms (SNPs) are usually not the functional variants driving the association and, in many cases, regional linkage disequilibrium (LD) prevents identification of a single candidate gene in the region. Often, additional studies are required to demonstrate unambiguously that the gene and/or variant implicated in disease risk is functionally related to pathogenesis.

Recently, the International Genomics of Alzheimer's Project (IGAP) identified 11 new loci (*p* < 10^−8^) associated with risk for Alzheimer's disease (AD), and 13 additional suggestive loci (*p* value between10^−6^ and 10^−8^) (Lambert et al., 2013). Among the latter group, there is an inter-genic SNP (rs9381040; *p* < 6.3 × 10^−7^) located 5.5 Kb downstream from *TREML2* and 24 Kb upstream from *TREM2*. The *TREM* and *TREM-like* *receptor* genes clustered on chromosome 6p21.1 (Ford and McVicar, 2009) have different patterns of LD among them (Cruchaga et al., 2013). This genomic region has previously been implicated in genetic risk for AD (Benitez et al., 2013, Bertram et al., 2013, Cruchaga et al., 2013, Guerreiro et al., 2013, Jonsson et al., 2012, Reitz and Mayeux, 2013). A low frequency missense variant in *TREM2* (p.R47H, minor allele frequency = 0.003) was reported to substantially increase risk for AD (Benitez et al., 2013, Guerreiro et al., 2013). SNPs in this region were also found to be associated with a cerebrospinal fluid (CSF) biomarker for AD (phospho-tau_181_ levels) (Cruchaga et al., 2013). Because of the design of the IGAP study (a meta-analysis) and the low frequency of the *TREM2* variant, it was not possible to determine whether the GWAS signal of this variant (rs9381040) was independent of the *TREM2*-p.R47H variant. In this study, we used exome-sequencing data to identify the most likely functional variant in *TREML2* responsible for the GWAS signal and to determine whether this signal is independent of *TREM2*-p.R47H (rs75932628) variant.

## 2 Methods

### 2.1 Exome sequencing Knight-Alzheimer's Disease Research Center (ADRC)

Enrichment of coding exons and flanking intronic regions was performed using a solution hybrid selection method with the SureSelect human all exon 50 Mb kit (Agilent Technologies, Santa Clara, CA, USA) following the manufacturer's standard protocol on 46 unrelated AD cases and 39 unrelated controls from the Knight-ADRC. This was performed by the Genome Technology Access Center at Washington University in St Louis (https://gtac.wustl.edu/). The captured DNA was sequenced by paired-end reads on the HiSeq 2000 sequencer (Illumina, San Diego, CA, USA). Raw sequence reads were aligned to the reference genome National Center for Biotechnology Information (NCBI) 36/hg18 by using Novoalign (Novocraft Technologies, Selangor, Malaysia). Base and/or SNP calling was performed using SNP SAMtools (Li et al., 2009). SNP annotation was carried out using version 5.07 of SeattleSeq Annotation server (see URL) (Benitez et al., 2011). On average, 95% of the exome had fold coverage >8.

### 2.2 UK-National Institute on Aging (UK-NIA) Dataset

A description of the UK-NIA dataset can be found in Guerreiro et al. (2013). Briefly, this dataset includes whole-exome sequencing data from 143 AD cases and 183 controls (Table 1).

### 2.3 Alzheimer's disease genetic consortium methods

Data used in the preparation of this article were obtained from the Alzheimer's disease genetic consortium (ADGC). A description of the samples included in the study as well as the methods used can be found in Naj et al. (2011). Imputed data from 10,067 AD cases and 9606 controls from the ADGC were used in this study (Naj et al., 2011). Genome-wide imputation was performed per cohort using MACH software with HapMap phase 2 (release 22) CEPH Utah pedigrees reference haplotypes and genotype data passing quality control as inference. Imputation quality was determined as r^2^ and only SNPs imputed with r^2^ ≥ 0.50 were included in the analysis. A multivariate logistic regression was performed to evaluate the association between genetic markers and risk for late-onset AD (LOAD) adjusting for age, gender, population substructure, and study-specific effects.

### 2.4 For use of genetic and environmental risk for Alzheimer's disease genotype data from “the 610 group”

Data used in the preparation of this article were obtained from the Genetic and Environmental Risk for Alzheimer's disease (GERAD) Consortium. The imputed GERAD sample comprised 3177 AD cases and 974 healthy elderly (age >70) control subjects with available age and gender data. Cases and elderly screened control subjects were recruited by the Medical Research Council (MRC) Genetic Resource for AD (Cardiff University; Institute of Psychiatry, London; Cambridge University; Trinity College Dublin), the Alzheimer's Research UK Collaboration (University of Nottingham; University of Manchester; University of Southampton; University of Bristol; Queen's University Belfast; the Oxford Project to Investigate Memory and Ageing, Oxford University); Washington University, St Louis, United States; medical research council PRION Unit, University College London; London and the South East Region AD project, University College London; Competence Network of Dementia, and Department of Psychiatry, University of Bonn, Germany; the National Institute of Mental Health AD Genetics Initiative. A number of 6129 control subjects were drawn from large existing cohorts with available GWAS data, including the 1958 British Birth Cohort (http://www.b58cgene.sgul.ac.uk), the KORA F4 Study and the Heinz Nixdorf Recall Study. All AD cases met criteria for either probable (National Institute of Neurological and Communicative Disorders and Stroke and the Alzheimer's Disease and Related Disorders Association [NINCDS-ADRDA], Diagnostic and Statistical Manual of Mental Disorders [DSM-IV]) or definite (Consortium to Establish a Registry for Alzheimer's Disease [CERAD]) AD. All elderly controls were screened for dementia using the MMSE or ADAS-cog, were determined to be free from dementia at neuropathological examination or had a Braak score of 2.5 or lower. Genotypes from all cases and control subjects were previously included in the AD GWAS by Harold et al. (2009). Imputation of the dataset was performed using IMPUTE2 and the 1000 genomes (http://www.1000genomes.org/) Dec2010 reference panel (NCBI build 37.1). The imputed data was then analyzed using logistic regression including covariates for country of origin, gender, age, and 3 principal components were obtained with EIGENSTRAT (EIGENSOFT 4.2) (Patterson et al., 2006) software based on individual genotypes for the GERAD study participants.

### 2.5 European Alzheimer's disease initiative consortium

All AD cases were ascertained by neurologists from Bordeaux, Dijon, Lille, Montpellier, Paris, Rouen, and were identified as French Caucasian (Dreses-Werringloer et al., 2008, Group, 2003). Clinical diagnosis of probable AD was established according to the DSM-III-R and NINCDS-ADRDA criteria. Control subjects were selected from the 3C Study (Group, 2003). This cohort is a population-based, prospective (7-years follow-up) study of the relationship between vascular factors and dementia. It has been carried out in 3 French cities: Bordeaux (southwest France), Montpellier (southeast France), and Dijon (central eastern France). A sample of non-institutionalized, over-65 subjects was randomly selected from the electoral rolls of each city. Between January 1999 and March 2001, 9686 subjects meeting the inclusion criteria agreed to participate. After recruitment, 392 subjects withdrew from the study. Thus, 9294 subjects were finally included in the study (2104 in Bordeaux, 4931 in Dijon, and 2259 in Montpellier). Genomic DNA samples 38 of 7200 individuals were transferred to the French Centre National de Génotypage. First stage samples that passed DNA quality control were genotyped with Illumina Human 610-Quad BeadChips (n = 452). At the end, we removed 308 samples because they were found to be first- or second-degree relatives of other study participants, or were assessed non-European descent based on genetic analysis using methods described in Heath et al. (2008). In this final sample, at 7 years of follow-up, 459 individuals suffered from AD with 97 prevalent and 362 incident cases. These AD cases were included as cases in the European Alzheimer's disease initiative (EADI) discovery dataset. We retained the other individuals as control subjects (n = 6017). The imputation was performed using 1000 Genomes multi-ethnic data (1000 G phase 1 integrated variant set release v3) as reference panel. Imputation was performed in 2 steps: pre-phasing with SHAPEIT (v2), followed by imputation with IMPUTE2. SNPs are used in the imputation process if call rate >98%, Hardy-Weinberg equilibrium (HWE) *p* value > 1e-6, minor allele frequency (MAF) > 1.

### 2.6 CSF levels dataset

A description of the CSF dataset used in this study can be found in Cruchaga et al. (2013) and data included 1269 unrelated individuals recruited through the Knight-ADRC at Washington University (n = 501, 73% CDR = 0), the Alzheimer's Disease Neuroimaging Initiative (n = 394, 27% Clinical Dementia Rating [CDR] = 0), a biomarker consortium of Alzheimer disease centers coordinated by the University of Washington (n = 323, 61% CDR = 0), and the University of Pennsylvania (UPenn) (n = 51, 2% CDR = 0). Briefly, CSF tau, phospho-tau-181 (ptau), and amyloid beta (Aβ_42_) levels were from research participants enrolled in longitudinal studies at the Knight-ADRC, ADNI, University of Washington, and University of Pennsylvania. CSF collection and Aβ_42_, tau, and ptau181 measurements were performed as described previously (Fagan et al., 2006). The samples were genotyped using Illumina chips. Cases received a diagnosis of dementia of the Alzheimer's type, using criteria equivalent to the National Institute of Neurological and Communication Disorders and Stroke-Alzheimer's Disease and Related Disorders Association for probable AD (McKhann et al., 1984). Controls received the same assessment as the cases but were nondemented. All individuals were of European descent and written consent was obtained from all participants.

### 2.7 Statistical analyses

We performed multivariate logistic regression to evaluate the association between genetic markers and risk for LOAD adjusting for age, gender, population substructure, and study specific effects using PLINK (http://pngu.mgh.harvard.edu/purcell/plink/). Conditional analysis was performed to identify additional independent signals by conditioning on the top case-control GWAS hits. We first estimated the odds ratios for SNPs across cohorts. These models calculate crude odds ratios and confidence intervals from counts of heterozygous in case patients and control subjects in each study. Then we performed a fixed-effect model to combine the odds ratios from study-specific estimates into a summary measure. No multiple-testing correction was used in our analyses. The heterogeneity of effects was evaluated using Woolf test for heterogeneity (Woolf, 1955). Meta-analysis was conducted using the META package (http://www.stats.ox.ac.uk/∼jsliu/meta.html) in R (version 3.0.1).

Association of CSF ptau with the genetic variants was analyzed as described previously (Cruchaga et al., 2010, Cruchaga et al., 2011, Kauwe et al., 2011). Briefly, CSF ptau values were log transformed to approximate a normal distribution. Because the CSF levels were measured using different platforms (Innotest plate ELISA vs. AlzBia3 bead-based ELISA, respectively), we were not able to combine the raw data. We extracted from the log-transformed value, the mean within each series for the log-transformation. No significant differences in the transformed CSF values of the different series were found. We used SAS (version 9.2) to analyze the association of SNPs with CSF biomarker levels. Age, gender, site, and the first 3 principal components were included as covariates. We also performed conditional analyses by including several variants in the model.

### 2.8 Genotyping

rs9381040 and rs3747742 were extracted from the GWAS data (Cruchaga et al., 2013), and confirmed by direct genotyping. The *TREM2*-p.R47H was genotyped using KASP genotyping assay (LGC Genomics), as previously described (Benitez and Cruchaga, 2013, Cruchaga et al., 2009, Cruchaga et al., 2010, Cruchaga et al., 2012) on 2000 cases and control subjects from the Knight-ADRC.

### 2.9 Cell-based analysis

Primary astrocytes and microglia were prepared from 2 litters (16 pups) of P1 C57BL/6 mice. Individual mice were pooled and 12 replicate co-cultures were plated in 25 cm^2^ flasks. Co-cultures were treated with 0.2 ng/mL of mouse interleukin-1 beta (IL-1β) (R&D 401-ML/CF) for 24 hours. Microglia was detached from the plate by shaking at 125 rpm for 1 hour in a 37 °C incubator. RNA was extracted using MiRNeasy mini kit (Qiagen 217004), according to manufacturer's instructions. The quantitative polymerase chain reaction assays for mouse Trem2 (ID: Mm04209424), Treml2 (ID: Mm01277362), and Saa3 (ID: Mm00441203) were obtained from Life Technologies (NY, USA).

## 3 Results

**Fig. 1:**
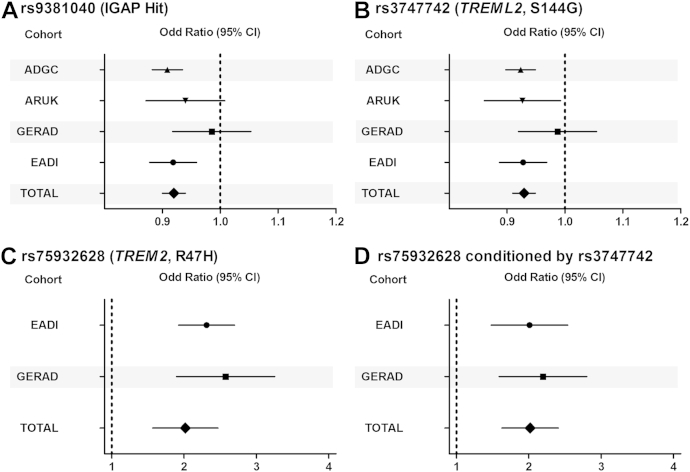
Odds ratios for rs9381040 (IGAP hit), rs3747742 (*TREML2*, p.S144G), and rs75932628 (*TREM2*, R47H) among AD patients, as compared with control subjects, at each study center and overall. Shown are the combined estimates of the AD risk of possessing rs9381040 (IGAP hit), combined odds ratios analyses were homogeneous (*p* = 0.69, by Woolf test for heterogeneity). Panel (A), the rs3747742 (*TREML2*, p.S144G) (*p* = 0.81, by Woolf test for heterogeneity), panel (B), the rs75932628 (*TREM2*, p.R47H) (*p* = 0.97, by Woolf test for heterogeneity), panel (C), rs75932628 (*TREM2*, p.R47H) after conditioning for rs3747742 (*TREML2*, p.S144G) panel (D). The triangles represent ADGC study, the inverted triangles represent ARUK study, squares represent GERAD study, circles represent EADI study and the diamonds represent the summary odds ratio. The horizontal lines indicate the 95% confidence intervals of the estimates. Abbreviations: ADGC, Alzheimer's disease genetic consortium; ARUK, Alzheimer's Research UK; EADI, European Alzheimer's disease initiative; IGAP, international genomics of Alzheimer's project; GERAD, genetic and environmental risk for Alzheimer's disease.

Eight coding variants were validated in the *TREML2* gene (Table 1), which constitute the 53% (8/15) of the missense variants reported for *TREML2* gene in the Exome Variant server (release ESP6500SI-V2) for European Americans. Only 3 variants exhibit a MAF % higher than 1%: p.V25A (MAF = 5%), p.T129S (MAF = 4.5%), and p.S144G (MAF = 30%). Interestingly, according to our exome sequencing results all these variants are more common in control subjects than in AD cases, however they did not reach statistical significance with our whole-exome sequence sample size, although the three of them are more common in control subjects than AD cases (Table 1). Interestingly, the missense variant p.S144G (rs3747742) exhibited the highest LD (r^2^ = 0.73, D′ = 0.86) with the GWAS SNP, rs9381040 (Table 1), and the higher MAF among the validated missense variants in *TREML2,* which made it suitable for further analysis. Next, we performed a meta-analysis of the data from the ADGC, GERAD, EADI, and the Alzheimer's Research UK; studies (16,254 cases and 20,052 control subjects) we found that the minor alleles of both rs9381040 (*p* = 1.21 × 10^−5^; OR = 0.92, CI = 0.88–0.95), and rs3747742 (*p* = 8.66 × 10^−5^; OR = 0.93, CI = 0.89–0.96) reduce risk for AD (Fig. 1, panel A and B). When rs3747742 is included in a logistic regression model as a covariate, rs9381040 is no longer significant (*p* = 0.43), and vice-versa, indicating that these SNPs are tagging the same signal. In addition, *TREM2*-p.R47H (rs75932628) was successfully imputed (imputation quality score information = 0.84 and 0.79) in the GERAD and EADI studies, and it displays a strong association with AD risk (*p* = 1.3 × 10^−3^; OR = 1.92, CI = 1.29–2.85) (Fig. 1, panel C). When rs3747742 or rs9381040 are included as covariates in a conditional analysis, rs75932628 remains highly significant (*p* = 1.27 × 10^−4^ and *p* = 1.19 × 10^−4^, respectively) (Fig. 1, panel D), suggesting that the *TREML2* and *TREM2* signals are independent from each other.

**Fig. 2:**
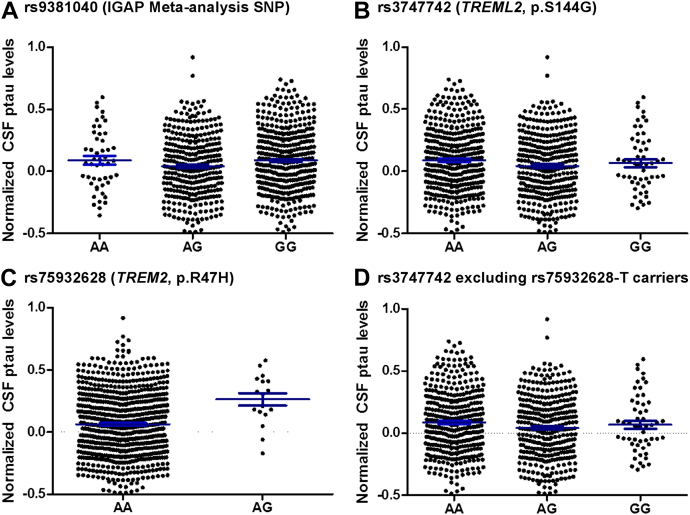
Association of *TREM2* and *TREML2* variants with CSF ptau levels. Panel (A) CSF ptau181 levels by rs9381040 genotype (IGAP meta-analysis most significant SNP). AG + GG versus AA *p* = 0.04. (Panel B) CSF ptau181 levels by rs3747742 genotype (*TREML2*, missense variant p.S144G). AG + GG versus AA *p* = 0.02. Panel (C) CSF ptau181 levels by rs75932628 genotype (*TREM2*, missense variant p.R47H). AG versus AA *p* = 0.0016. Panel (D) CSF ptau181 levels by rs3747742 genotype (*TREML2*, missense variant p.S144G). AG + GG versus AA excluding the variant p.R47H carriers *p* = 0.03. The mean and the standard error of the mean (SEM) for the normalized residuals CSF ptau181 levels are shown in blue. Abbreviations: CSF, cerebrospinal fluid; IGAP, international genomics of Alzheimer's project; SNP, single nucleotide polymorphisms.

We also performed a linear regression analysis for rs9381040 and rs3747742 with CSF levels of tau and ptau (n = 1269 individuals) (Cruchaga et al., 2013). rs9381040 (*p* = 4.11 × 10^−4^, beta = −0.02) and rs3747742 (*p =* 1.4 × 10^−4^, beta = −0.02) both exhibit a strong association with CSF ptau levels. The respective associations with CSF ptau are no longer significant when either SNP is included as a covariate in the conditional analysis. These results confirm via 2 independent datasets that the associations of rs9381040 and rs3747742 with CSF biomarker levels and with AD risk represent the same signal. The *TREM2*-p.R47H variant was also genotyped in a subset of the CSF samples (n = 835). In these samples, 3 variants, rs9381040 (*p* = 0.04, beta = −0.02) (Fig. 2, panel A), rs3747742 (*p* = 0.02, beta = −0.02) (Fig. 2, panel B), and rs75932628 (*p* = 0.0016, beta = 0.2) (Fig. 2, panel C) demonstrate a nominally significant association with CSF ptau levels. To determine whether the *TREML2* signal (rs3747742) is independent of *TREM2*-p.R47H, we removed all of the p.R47H carriers from the analysis. rs3747742 remained significantly associated with CSF ptau levels (*p* = 0.03) (Fig. 2, panel D). Furthermore, when *TREM2*-p.R47H was included in the model as a covariate for rs3747742 analysis, the association remained significant (*p* = 0.02), which suggests that the *TREM2* and *TREML2* signals are independent. Importantly, these associations confirmed the direction of the effect on CSF ptau levels: the minor allele of rs3747742 is associated with lower ptau levels (beta = −0.02) and is predicted to be protective for AD risk (OR = 0.91; CI = 0.86–0.97), while the minor allele of *TREM2*-p.R47H is associated with an increased risk for AD (OR = 1.91, CI = 1.85–1.97) and higher levels of CSF ptau (beta = 0.2).

**Fig. 3:**
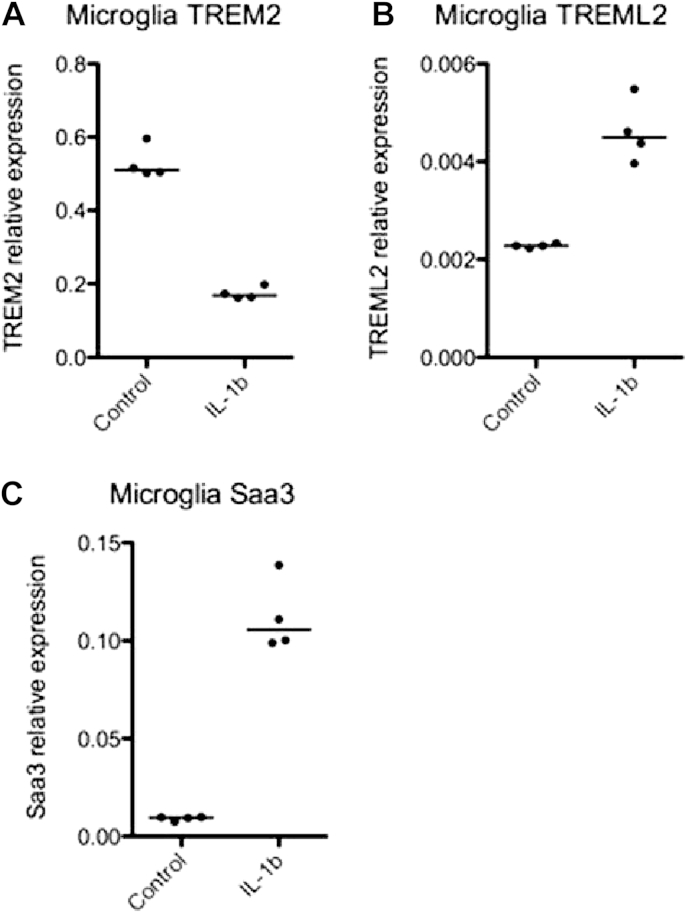
Microglial expression of *TREM2* and *TREML*2 show opposing effects in the presence of IL-1b. *TREM2* panel (A) and *TREML2* panel (B) gene expression were analyzed in primary mouse microglia and astrocytes activated by 0.2 ng/mL IL-1β for 24 hours. Induction of Saa3 expression panel (C) serves as a positive control for IL-1β stimulated activation. Abbreviation: IL-1β, interleukin-1 beta.

In addition, TREM and TREM-like receptors modulate the innate immune response by either amplifying or dampening Toll-like receptor-induced signals, playing critical roles in fine-tuning the inflammatory response (Ford and McVicar, 2009). TREM and TREM-like receptors demonstrate different patterns of expression and are likely to play different roles in the inflammatory response. To further understand the relative expression of *TREM2* and *TREML2,* we analyzed gene expression in primary mouse microglia and astrocytes stimulated by IL-1β. Treatment of microglia with IL-1β repressed expression of *TREM2* (Fig. 3, panel A), but increased expression of *TREML2* (Fig. 3, panel B). The opposing effects of this inflammatory cytokine on *TREM2* and/or *TREML2* expression is consistent with our genetic data and with evidence that *TREM2* and/or *DAP12* antagonizes inflammatory signaling in microglia while *TREML2* is not coupled to *DAP12* signaling and plays a pro-inflammatory role (Ford and McVicar, 2009).

## 4 Discussion

In summary, these results demonstrate that the associations of missense variants in *TREM2* and *TREML2* with AD risk are independent. Moreover, our analyses suggest that the AD-associated GWAS signal is likely driven by the *TREML2* coding missense variant p.S144G (rs3747742); it results in a similar odds ratio to rs9381040. We also validated 2 other coding variants p.V25A and p.S129T in *TREML2* gene in moderate LD (r^2^ = 0.05 and D′ = 1) with the GWAS SNP, which both exhibited a higher frequency among control subjects than in AD cases (Table 1). However, for both variants we only obtained data by whole-exome sequencing which limited our analysis about the role that these variants may play in the association of *TREML2* with AD risk. To prove that these additional variants are associated with AD risk we will need a larger sample size. Additionally, the purpose of this study was to find a functional coding variant in the *TREML2* gene that could explain the association for *TREML2* which was found in the recent IGAP meta-analysis. Our data suggest that there is a coding variant in *TREML2* that could explain the GWAS signal, but our data cannot rule-out of the presence of functional variants outside of the coding region.

We conclude that at least 2 genes in this gene cluster influence risk for AD: *TREM2*-p.R47H is associated with increased risk for AD (OR = 1.91, CI = 1.85–1.97) and *TREML2*-p.S144G is associated with reduced risk for AD (OR = 0.91; CI = 0.86–0.97). The mechanisms by which these variants influence AD risk are not currently understood, but it has been suggested that modulation of microglial activation might influence clearance of Aβ (Benitez et al., 2011). These results underline the importance of the inflammatory response in modulating risk for AD and suggest that other genes in this gene family may also harbor risk alleles for AD.

## Disclosure statement

The authors report no conflicts of interest.

All participants had agreed by signed informed consent to participate in genetic studies approved by our Institutional Review Board.

## Figures and Tables

**Table 1:** TREML2 variants identified by exome-sequencing

Location in chromosome 6	rs#	AA change	EVS, MAF	AD (n = 189)		Control subjects (n = 225)		OR (95% CI)	*p* value	LD with rs9381040		Condel	Sift	Polyphen
Hets	MAF	Hets	MAF	r^2^	D′
41166154	rs77704965	D23G	0.22	0	0%	4	2%	—	0.17	0.018	1	Neutral	Tolerated	Benign
41166149	rs62396355	V25A	5.05	6	3%	15	7%	0.45 (0.17–1.2)	0.11	0.018	1	Neutral	Tolerated	Benign
41166075	rs35512890	M50V	—	16	8%	27	12%	0.67 (0.35–1.3)	0.24	—	—	Neutral	Tolerated	Benign
41162562	rs61734887	S129T	4.52	12	6%	22	10%	0.62 (0.30–-1.3)	0.2	0.051	1	Neutral	Tolerated	Benign
41162538	—	L137H	—	0	0%	1	0%	—	0.35	—	—	Neutral	Tolerated	Benign
41162518	rs3747742	S144G	30.44	82	43%	104	47%	0.89 (0.6–1.31)	0.56	0.67	0.86	Neutral	Tolerated	Benign
41162371	rs145455750	T193A	0.27	0	0%	1	0%	—	0.35	—	—	Neutral	Tolerated	Benign
41162204	rs115991880	S248A	0.34	2	1%	5	2%	0.47 (0.09–2.45)	0.36	0	0	Deleterious	Deleterious	Benign

Coding variants in *TREML2* were extracted from 46 unrelated AD cases and 39 unrelated controls from the Knight-ADRC study and from 143 unrelated AD cases and 186 unrelated controls from the NIA-UK exome-sequencing study. The r^2^ and D′ values reported here are coming from the Pilot 1 of the 1000 K genome project.

Key: AD, Alzheimer's disease; CI, confidence interval; LD, linkage disequilibrium; OR, odds ratio.
